# The Finnish Interprofessional Medication Assessment (FIMA): baseline findings from home care setting

**DOI:** 10.1007/s40520-018-1085-8

**Published:** 2018-12-05

**Authors:** K. Auvinen, J. Räisänen, M. Merikoski, A. Mäntylä, A. Kumpusalo-Vauhkonen, H. Enlund, T. Liukkonen, J. Jyrkkä, E. Lönnroos, P. Mäntyselkä

**Affiliations:** 1 The East Savo Hospital District, BOX 111, 57101 Savonlinna, Finland; 2 Institute of Public Health and Clinical Nutrition, Faculty of Health Sciences, University of Eastern Finland, Kuopio, Finland; 3 Assessment of Pharmacotherapies, Finnish Medicines Agency, Kuopio, Finland; 4 Kärsämäki Pharmacy, Kärsämäki, Finland; 5 Vieremä Pharmacy, Vieremä, Finland; 6 The South Savo Hospital District, Mikkeli, Finland; 7 Primary Heath Care Unit, Kuopio University Hospital, Kuopio, Finland

**Keywords:** Aged, Older people, Home care, Interdisciplinary health team, Medicines, Geriatric assessment

## Abstract

**Purpose:**

Medication-related problems and declined functional capacity are closely associated factors among older people. The purpose of this study is to describe the procedure of interprofessional medication assessment in home care context and the baseline characteristics of the study population.

**Methods:**

The FIMA study was a randomized, controlled intervention study comparing general practitioner-led interprofessional medication assessment and usual care. Patients’ chronic diagnoses and medication use as well as physical and cognitive functions were investigated. Performance in daily activities, use of care services and help from family and relatives, self-rated health and health-related quality of life, and adverse effects commonly related to medication were assessed.

**Results:**

The home care patients (*n* = 512) had significant disease burden and functional limitations. The mean number of all medicines was 15 and that of regularly taken medicines 10. The majority of patients (87%) had excessive polypharmacy. The most commonly used (97%) ATC medicine class was nervous system medicines. Clinically relevant (class C or D SFINX record) drug–drug interactions were seen in 74% of the patients. The most frequent risks of adverse effects were risk of bleeding (66%), constipation (58%) and orthostatism (54%) occurring in over half of the patients. Medicines affecting renal function were used by 85% of the patients.

**Conclusions:**

There is an evident need and justification for medication assessments in home care. In most cases, home care patients fulfill the criteria for regular medication assessments.

## Background

Older age groups are the most rapidly growing segments of the population in Finland as in many European countries [1, 2]. Demographic aging leads to increasing need for different types of care services, including home-delivered care. The number of home care patients is rapidly increasing, and at the same time, they are older and frailer than previously [2, 3]. Consequently, maintaining functional capacity is a key prerequisite for living at home [4].

Aging is associated with multimorbidity and polypharmacy [5] which is related to the use of inappropriate medicines [6], functional decline and negative clinical outcomes [7], including drug-related adverse events [8]. Among older home care patients, both polypharmacy and the use of inappropriate medicines are common. In a large European study, 19.8% of home care patients used at least one inappropriate medicine and polypharmacy (≥ 6 medicines) was documented in 51% of the patients [9].

Numerous interventions to reduce polypharmacy and complex medications have been described [10]. Interprofessional team approach is suggested to be advantageous when assessing patients with multiple diseases and complex medications [11, 12]. However, the effect of medication assessments on clinically important outcomes and patient-centered outcomes is still unclear [10]. In Finnish home care, systematic medication reviews are seldom conducted, and pharmacist consultations are rare. Therefore, we conducted the Finnish Interprofessional Medication Assessment (FIMA) study to develop a clearly defined, repeatable, practice-based model of interprofessional medication assessment for home care settings. In general, the objective of the FIMA study was to examine the effects of interprofessional medication assessment (the FIMA model) on medication, functional capacity, quality of life, and use and costs of health and home care services in home care patients [13]. The aim of the present study was to define the sociodemographic characteristics, morbidity, functional capacity and medication use among home care patients of the FIMA study.

## Methods

### Setting

**Fig. 1:**
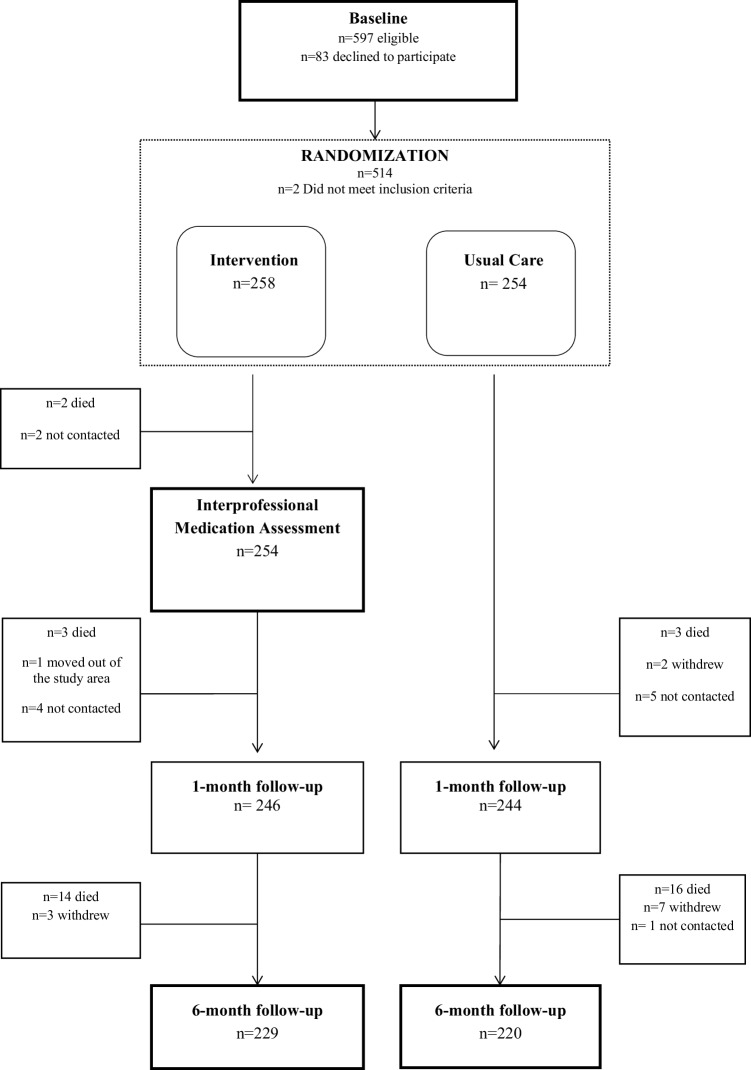
Flow chart of the Finnish Interprofessional Medication Assessment Study

The FIMA study was a randomized, controlled intervention study with comparison between general practice (GP)-led interprofessional medication assessment and usual care (Fig. 1). The FIMA study is registered at ClinicalTrials.gov (https://clinicaltrials.gov/ct2/show/NCT02398812). The study was conducted in public home care settings in five areas in Finland: Forssa, Haapajärvi, Lahti, Juva and Savonlinna. The centers were recruited from the interprofessional network constructed to establish guidelines for interprofessional collaboration in medication management of the aged, organized by the Finnish Medicines Agency (FIMEA) [14].

### Patients

We screened and recruited patients receiving regular home care services in the study areas. Inclusion criteria were age at least 65 years and registration with public home care services, and at least one of the following: currently taking ≥ 6 medicines daily, currently having dizziness, orthostatic hypotension, or experienced a fall in previous 12 months. We excluded patients whose medication was not managed by home care and patients with active cancer therapy.

The sample size was estimated using Timed Up and Go (TUG) as a primary outcome. We regarded TUG value of 8.3 s as a mean (SD 1.9) [15], and 1 s as a clinically significant difference between groups. To reject the null hypothesis for an error type I equal 5% and error type II of 20, a sample size of 114 was obtained. However, we predicted possible losses from follow-up and considered multiple outcomes. Therefore, we aimed to have at least 300 patients per group.

In total, 512 patients were recruited by home care nurses, and after baseline measurements from February to December 2015, they were randomized into intervention or usual care using block randomization with blocks of ten. The study assistant implemented the random allocation sequences and pharmacists assigned participants to intervention.

### Baseline data collection

Medication use was verified by a home care nurse who obtained patient’s current medication list from the electronic medical record before the baseline measurements. At patient’s home, the nurse checked prescription and over-the-counter (OTC) medicines including vitamins, mineral supplements and natural products the patient used, and updated the medication list accordingly.

The home care nurse interviewed the patient using structured questionnaires. Performance in daily activities, use of care services and help from family and relatives, self-rated health and health-related quality of life, and adverse effects commonly related to medication as well as patient’s physical performance were assessed. In addition, blood samples were taken for laboratory examinations (blood count, sodium, potassium, creatine kinase).

The home care team physician documented patients’ diagnoses from the existing medical records. Cardiovascular diseases included coronary artery disease, hypertension, chronic heart failure, atrial fibrillation and valvular heart diseases. Cerebrovascular diseases included stroke, transient ischemic attack, subarachnoid hemorrhage and intracranial hemorrhage. Diseases of the musculoskeletal system involved arthrosis, rheumatoid arthritis, gout and osteoporosis. Diabetes mellitus (type I and II) was recorded. Neurological diseases included Parkinson disease and epilepsy. Memory disorders were categorized as Alzheimer’s disease, vascular dementia, Lewy body dementia, frontotemporal dementia, mixed dementia and other or nonspecified dementias. Asthma and chronic obstructive pulmonary disease were recorded. Psychiatric conditions included depression, psychosis and neurotic disorders. History of cancer, current stage and primary organ were recorded. History of gastrointestinal bleeding was recorded. We used modified Charlson Comorbidity Index (CCI) [16] to describe home care patients’ disease burden. The index was calculated using the following diseases with corresponding scores: metastatic or terminal cancer (score of 6); non-metastatic cancer and moderate or severe renal insufficiency (score of 2); heart failure, coronary artery disease, type 1 or 2 diabetes, chronic asthma or chronic obstructive pulmonary disease, rheumatoid arthritis or other forms of inflammatory arthritis, peripheral vascular disease, cerebrovascular disease, dementia of any type or history of gastrointestinal bleeding (score of 1).

### Measurements

Katz index of activities of daily living (ADL) [17] and the Lawton and Brody scale of Instrumental Activities of Daily Living (IADL) scale [18] were used to assess patients’ performance. The ADL index involves basic activities such as body care, dressing, toileting, transferring and feeding, while the IADL scale covers complex instrumental activities, such as using the telephone, shopping, preparing meals, cleaning, washing clothes, using public transport and managing medication and finances. All items in the ADL and IADL questionnaires are dichotomous, 1 indicating ability to conduct the task and 0 indicating disability. Maximum score in ADL is 6 and in IADL 8, with lower scores indicating increased require for assistance in daily activities. ADL scores 5–6 indicate normal or mild dependency, 3–4 indicate moderate and ≤ 2 severe dependency [19].

TUG test was used to assess mobility, lower extremity strength and balance. Patients are timed (in seconds) while rising from a seated position in a chair with armrests, walking 3 m, turning around, walking back and sitting down. The time taken to complete the TUG test correlates with level of functional mobility [20]. In this study, we use the cut-off score ≥ 13.5 s, that identifies individuals classified as high fall risk [21].

The mini-mental state examination (MMSE) was used for screening cognitive function. MMSE is a standard tool that is widely used to screen cognitive disorders particularly in older people [22]. The MMSE sum scores from 30 to 25 were categorized as normal while scores from 24 to 19 indicated mild, 18–12 moderate and ≤ 11 severe cognitive impairment [23].

Geriatric Depression Scale (GDS-15) was used for assessing depressive symptoms. GDS-15 is a 15-item screening tool to measure depressive symptoms in older adults. The items of GDS-15 represent characteristics of depression in the affective and cognitive domains. Sum scores ≥ 6 are suggestive of depression and scores ≥ 11 indicate high likelihood for depression [24, 25].

The preference-based, five-dimension instrument provided by EuroQol (EQ-5D) was used for measuring health-related quality of life. The EQ-5D-3L addresses five different dimensions of health: (1) mobility; (2) self-care; (3) usual activities; (4) pain/discomfort; and (5) anxiety/depression. Three answer levels are provided for each item with the first level referring to the best state. The information derived from the EQ-SD self-classifier is converted into a single summary index by applying scores from Finnish valuation sets [26]. Utility scores range from − 0.590 to 1, where 1 indicates perfect health, 0 indicates death, and values below 0 indicate health states worse than death. The visual analogue scale (VAS) records patients’ perception of their overall health on a scale from 0 to 100 with 0 denoting the worst and 100 the best imaginable health state [27, 28].

Orthostatic hypotension (OH) was defined as fall in systolic blood pressure ≥ 20 mmHg and/or diastolic blood pressure ≥ 10 mmHg within 3 min. The blood pressure and heart rate measurements are obtained after the patient has been supine for 15 min, right after getting to sitting position, and after 1 min and 3 min of standing [29, 30]. In addition, notation should be made of any symptoms that the patient experiences upon standing.

The glomerular filtration rate (GFR) was calculated with the CKD-EPI formula [31]. In this study, GFR ≤ 50 mL/min was regarded as moderate renal insufficiency and GFR 51–80 mL/min as mild renal insufficiency.

The number of medicines was classified as polypharmacy (6–9 medicines) and excessive polypharmacy (10 or more medicines). Medicines were classified according to the Anatomical Therapeutic Chemical Classification System (ATC). In the ATC classification, the medicines are divided into groups according to the organ or system on which they act and their chemical, pharmacological and therapeutic properties [32].

### Intervention

Patients’ updated and verified medication lists and health measurements were used in the assessment. An interprofessional team consisting of a pharmacist, licensed physician and registered nurse regularly working in home care conducted the structured assessment within 2 weeks after baseline measurements. All pharmacists had a qualification in comprehensive medication review (CMR) or current continuing professional development in clinical pharmacy. The physician made clinical decisions and recommendations at the end of the team meetings. The nurse updated patient’s medication regimen and informed the patient about the changes, or if necessary, the patient participated in the interprofessional team meeting. All interprofessional team members received a 1-day training or a personal introduction concerning the FIMA study.

The pharmacist reviewed the patient’s medication list using the SFINX (currently INXBASE), Pharao (currently RISKBASE) and RENBASE databases [33]. SFINX is a drug–drug interaction (DDI) database, which classifies interactions into classes A–D based on clinical significance. Pharao presents a risk profile of patients’ medicines based on pharmacodynamic properties. RENBASE includes medicine-related information on safety and dosage with regard to renal function.

### Control

Patients randomized into the control group continued in usual home care (Fig. 1). The data were collected similarly in the intervention and the control groups. In addition, the pharmacist analyzed the medication lists using the same databases as with the intervention group. This review of medication lists occurred only after the 6-month measurements were conducted.

### Follow-up

One month after the baseline measurements, the home care nurse checked the patients’ medication use similarly to the baseline (Fig. 1). At 6 months, all the measurements conducted at baseline were repeated, including blood samples. This occurred between September 2015 and May 2016.

### Statistical analysis

The data were analyzed according to randomization group irrespective of whether or not the patients received the intervention as planned (the intention to treat principle).

The characteristics of the patients were summarized using percentages, means and standard deviations. Between groups, mean values were compared using two sample *t* tests for independent samples and differences in proportions were compared using a *χ*^2^ test. The significance of the results is presented as *p* values, and values of less than 0.05 are considered statistically significant. All statistical analyses were performed using IBM SPSS software version 24 (SPSS Inc, Chicago, Ill).

## Results

In general, there were no sociodemographic or clinical differences between the intervention and usual care groups at the beginning of the study (Table 1). The mean age of all the 512 home care patients was 84 years. The majority of all patients were women and living alone. Thirty-six percent of the patients had orthostatic hypotension. Most (81%) of the patients had at least mild renal insufficiency. The average CCI was 2.5 and the average number of chronic diseases per patient was 6.3. Of the recorded chronic conditions, cardiovascular diseases were the most common, followed by diseases of the musculoskeletal system, diabetes, cerebrovascular diseases and diagnosed dementia of any type. The mean ADL and IADL scores indicated that the patients were dependent in one of the six basic activities and four of the eight instrumental activities of daily living. Of all patients, 64% had an MMSE score indicative of cognitive impairment (< 25) while 38% had a GDS-15 score ≥ 6 suggestive of depression. Among all participants, the mean EQ-5D was 0.58 and the mean VAS score was 57. In the TUG test, 83% of the patients exceeded the cut-off time (13.5 s) for high risk of falls.

**Table 1:** Baseline characteristics for intervention (*n* = 258) and usual care (*n* = 254) groups

	Intervention *n* = 258	Usual care *n* = 254	*p* value
Age (years), mean (SD)	83 (6.7)	84 (6.2)	0.650
Female, *n* (%)	177 (68)	191 (75)	0.131
Living alone, *n* (%)	202 (78)	189 (75)	0.301
Orthostatic hypotension, *n* (%)	81 (34)	92 (39)	0.279
GFR (ml/min), mean (SD)	63 (19)	60 (18)	0.083
≤ 50 ml/min, *n* (%)	75 (29)	70 (28)	0.704
Chronic diseases, *n* (%)			
Cardiovascular diseases	234 (92)	237 (92)	0.979
Diseases of musculoskeletal system	158 (62)	155 (61)	0.872
Diabetes	91 (35)	92 (36)	0.874
Cerebrovascular diseases	79 (31)	81 (32)	0.904
Dementia	84 (33)	73 (29)	0.421
Respiratory diseases	52 (20)	43 (17)	0.538
Psychiatric diseases	49 (19)	39 (15)	0.058
Cancer	46 (18)	33 (13)	0.120
Gastrointestinal diseases	41 (16)	36 (14)	0.344
Neurological diseases	36 (14)	32 (13)	0.627
Charlson comorbidity index mean (SD)	2.6 (1.6)	2.4 (1.6)	0.130
0 *n* (%)	18 (7.0)	20 (7.8)	0.552
1–2 *n* (%)	111 (43)	124 (48)	
3–4 *n* (%)	97 (38)	86 (34)	
≥ 5 *n* (%)	32 (12)	26 (10)	
Functional capacity			
ADL, mean (SD)	5.0 (1.3)	4.9 (1.2)	0.145
5–6 *n* (%)	118 (46)	95 (37)	0.137
3–4 *n* (%)	124 (48)	144 (57)	
≤ 2 *n* (%)	16 (6.2)	15 (5.9)	
IADL, mean (SD)	4.1 (2.0)	4.2 (2.1)	0.986
Median (IQR)	4 (3,6)	4 (2,6)	
TUG, seconds, mean (SD)	30 (28)	26 (16)	0.143
Cognitive capacity			
MMSE, mean (SD)	22.9 (4.1)	23.1 (4.6)	0.469
30–25 *n* (%)	113 (44)	123 (49)	0.642
24–19 *n* (%)	110 (43)	96 (38)	
18–12 *n* (%)	27 (11)	28 (11)	
≤ 11 *n* (%)	7 (2.7)	5 (2.0)	
Depressive symptoms			
GDS-15, mean (SD)	5.4 (3.2)	5.0 (3.1)	0.085
Health-related quality of life			
EQ-5D score, mean (SD)	0.58 (0.25)	0.59 (0.25)	0.813
Median (IQR)	0.62 (0.52, 0.73)	0.62 (0.52, 0.73)	
EQ-5D VAS, mean (SD)	58 (17)	56 (18)	0.455
Median (IQR)	55 (49,70)	55 (49,70)	

*GFR* glomerular filtration rate, *ADL* activities of daily living, *IADL* instrumental activities of daily living, *MMSE* mini-mental state examination, *GDS-15* geriatric depression scale, *TUG* timed up&go test, *EQ-5D* EuroQol health-related quality of life, *VAS* visual analogue scale

Among the home care patients, the mean number of all medicines was 15 and that of regularly taken medicines was 10 (Table 2). The majority of patients (87%) had excessive polypharmacy (≥ 10 medicines). The three most commonly used ATC medicine classes were medicines for the nervous system (97%), cardiovascular system (96%) and for the alimentary tract and metabolism (91%). The average number of nervous system medicines per patient was 2.7.

**Table 2:** Medication use and clinically relevant interactions, risks of adverse effects and medicines affecting renal function

	Intervention *n* = 258	Usual care *n* = 254	*p* value
All medicines^a^, mean (SD)	15 (5.2)	15 (5.1)	0.234
Regularly taken	10 (3.5)	10 (3.1)	0.802
Taken as needed	4.5 (3.3)	4.4 (2.9)	0.831
Number of medicines^b^*n* (%)			
< 6	1 (0.4)	1 (0.4)	0.991
6–9	38 (15)	24 (9.4)	0.067
≥ 10	219 (57)	229 (90)	0.071
Drug–drug interactions^c^, *n* (%)	181 (70)	198 (78)	0.079
Risk of adverse effects^d^, *n* (%)			
Bleeding	161 (62)	179 (71)	0.072
Constipation	146 (57)	153 (60)	0.465
Orthostatic hypotension	133 (52)	144 (57)	0.258
Anticholinergic effect	73 (28)	80 (32)	0.464
Sedation	49 (19)	65 (26)	0.081
QT-prolongation	36 (14)	35 (14)	0.926
Serotonergic effect	5 (1.9)	9 (3.5)	0.272
Seizures	1 (0.4)	3 (1.2)	0.312
Medicines affecting renal function^e^, *n* (%)	210 (81)	223 (88)	0.034

^a^Including prescription and over-the-counter medicines

^b^Including regularly taken medicines and medicines taken as needed

^c^Class C and D interactions based on SFINX database

^d^Class C and D adverse effects based on PHARAO database

^e^Class C and D risks of drug-induced impairment of renal function based on RENBASE database

Three out of four patients (74%) had clinically relevant (class C or D SFINX record) drug–drug interactions. The most frequent risks of adverse effects were risk of bleeding (66%), constipation (58%) and orthostatism (54%) occurring in over half of the patients. In addition, the use of medicines with anticholinergic (30%) or sedative (20%) properties was also common. The risk of drug-induced impairment of renal function (class C or D RENBASE records) was present in 85% of the patients and the mean number of medicines with renal risks was 2.4 per patient.

## Discussion

This study highlighted the importance of medication assessment among home care patients. The home care patients had significant disease burden and functional limitations. A vast majority of the patients had excessive polypharmacy, significant DDIs and risks of adverse effects. Nearly all patients used central nervous system (CNS) active medicines. In this study, two-thirds of the patients had cognitive impairment according to MMSE scores.

In this study, the mean EQ-5D score and VAS were 0.58 and 57, respectively. The mean EQ-5D scores were reported to range from 0.45 to 0.78 in Finnish population aged 65 and older [34]. In another Finnish study concerning health-related quality of life in patients of multidisciplinary pain clinics, the mean EQ-5D score was 0.53 [35]. Our findings were thus concordant with previous Finnish studies.

In the present study, the prevalence of excessive polypharmacy was high (87%). In the large European Ad-HOC study, 22% of all homecare patients used ≥ 9 medicines while among the Finnish participants the share was almost twice as high, 41% [9]. The prevalence of excessive polypharmacy in previous Finnish studies [36, 37] has ranged from 41% (including all prescription- and OTC-medicines) to 55% (including regularly used prescription medicines). However, due to differences in study populations, direct comparisons to our findings are challenging. The former study was population based and concerned people aged ≥ 75 years, and the latter investigated people living in assisted aged care facilities, while our study involved home care patients using ≥ 6 medicines.

The rate of clinically relevant DDIs in our study was 190 per 100 patients. The corresponding rate ranged from 22 to 187 per 100 in a recent review of older primary care patients [38]. Up to 7.2% of our home care patients had class D DDIs. The prevalence of class D DDIs was 5.9% among residents living in aged care facilities [36] and 4.8% among nursing home patients in Finland [39].

Older people with cognitive impairment are at considerable risk of medication-related adverse effects [40]. Nervous system medicines were the most frequently prescribed in our study. The use of nervous system medicines was known to be common (62–73%) in Finnish older populations [41], but in our study the prevalence was even higher (97%) than reported previously.

One-third of our patients had orthostatic hypotension. Furthermore, the average TUG time (27 s) among all the patients indicated a substantial risk of falls [20, 21]. Both orthostatic hypotension and impaired gait are associated with risk of falls [42]. Depending on comorbidities and medications, the prevalence of orthostatic hypotension in people aged ≥ 65 years has been reported to range from 10 to 65% [43, 44].

Over 80% of our home care patients used medicines affecting renal function. This is a high proportion when compared to findings among outpatients with renal insufficiency [45]. However, our findings are close to prevalence reported for hospitalized patients with renal insufficiency [46].

Our study had several strengths. The FIMA was a randomized, controlled study in a real-life context. We used validated measurements to examine patients’ daily performance, functional and cognitive capacity, and quality of life. The detection and assessment of medicine-related risks and interactions were based on three decision support systems that are available and commonly used in Finnish health care. The advantages of the SFINX-PHARAO and RENBASE databases are that they contain constantly updated concise and evidence-based information on a wide number of medicines in user-friendly online format.

The FIMA procedure used in the present study differs from most of the previous medication reviews or assessments [47, 48] in three significant ways. First, the interprofessional team is GP led, and the pharmacist is a permanent member of the team. Significant information concerning patient’s clinical condition and functional capacity may be missed if the medication assessment is isolated from health care, as in many pharmacist-led assessments. In the FIMA procedure, the clinical pharmacist can target the review to clinically relevant problems and the physician is able to make changes to patient’s medication at the team meeting when all the information is available. Nurses receive instructions for patients’ further follow-up in the interprofessional team meetings, which reduces the risk for information disconnections. Second, the selection of the patients is not emphasized in the FIMA procedure, and this study shows clearly that home care patients usually fulfill previously defined criteria (polypharmacy, multimorbidity, inappropriate medication use) for medication assessment. Third, in contrast to comprehensive medication review (CMR) [49], the in-home interviews were conducted by nurses instead of pharmacists. This enables medication assessments for a large number of patients in routine care. The work in interprofessional team is more effective when all professionals have gathered information on patients beforehand. Nurses meet the patients regularly, and they are professional in collecting information on patient’s current health via in-home interviews which they can conduct as a part of their regular work. This offers crucial information for the pharmacist, who conducts the medication review and after this for the interprofessional team which makes the decisions on changes needed in the treatment of the patient. Nurses’ role is essential also after the interprofessional team work as they will accomplish the follow-up of the patient after the changes in the treatment.

The FIMA procedure does not cause extra work for home care nurses and physicians, because examining current medication, GFR, blood tests, blood pressure, orthostatic hypotension, cognitive function, depressive symptoms and capability for ADL and IADL functions is or should be the basic information that is needed for home care patients’ personal healthcare plans.

This study had some limitations. Despite training of the team and written instructions, there might have been differences in data collection and conduction of the measurements. In addition, the same interprofessional teams examined patients from intervention and control groups. The intervention was performed only once for each patient in the intervention group although medication assessments should be a regular procedure in home care settings.

## Conclusion

Our findings indicate that there is an evident need and justification for medication assessments in home care. In most cases, home care patients fulfill the criteria for regular medication assessments. The FIMA procedure is feasible and easy to conduct in home care settings. In addition, the skills of the professionals are utilized in an optimal and efficient way.
